# Endoscopic Retrieval of Retained Irrigation Tubing from a Barnett Continent Intestinal Reservoir

**DOI:** 10.7759/cureus.112314

**Published:** 2026-07-09

**Authors:** Marissa C Kuo, Christian Schuetz, Paul C Kuo, Alfredo C Cordova

**Affiliations:** 1 Department of Surgery, Vanderbilt University Medical Center, Nashville, USA; 2 Department of Surgery, Sarasota Memorial Hospital, Sarasota, USA; 3 Department of Surgery, University of South Florida Morsani College of Medicine, Tampa, USA

**Keywords:** barnett continent ileostomy reservoir, continent ileostomy, endoscopic retrieval, ileoscopy, retained irrigation tubing

## Abstract

Continent ileostomy, including Kock pouch and Barnett continent ileostomy reservoir (BCIR), is an alternative to conventional (Brooke) ileostomy following total colectomy. Because these procedures are performed infrequently and primarily at specialized centers, many providers may have limited familiarity with their anatomy and management. We present the case of a 61-year-old man with a history of ulcerative colitis who underwent total abdominal colectomy with BCIR creation. He presented after accidental retention of irrigation tubing within the pouch during routine catheterization. Gastroenterology was consulted for ileoscopy; however, endoscopic evaluation was initially deferred because of concern regarding the small caliber and atypical appearance of the continent stoma. The patient was brought to the operating room, where digital examination of the stoma demonstrated a patent stoma without evidence of stenosis or stricture, and endoscopic access through the stoma was achieved without difficulty. Approximately 30 cm of retained irrigation tubing was successfully removed using an endoscopic grasper. This case highlights an unusual complication of continent ileostomy catheterization and demonstrates that endoscopic access through a BCIR stoma may be feasible and safe in appropriately selected patients. Increased familiarity with continent ileostomy anatomy may help reduce uncertainty in management and avoid delays in intervention.

## Introduction

Continent ileostomies are an alternative to conventional end ileostomies for patients requiring total colectomy, most commonly in the setting of ulcerative colitis or familial adenomatous polyposis [1]. The technique involves the creation of an internal reservoir for bowel contents such that patients do not have to wear an external appliance; the intent is to improve quality of life by providing control over bowel movements through routine catheterization [2,3]. The Kock pouch is the most widely recognized continent ileostomy, while the Barnett continent intestinal reservoir (BCIR) was later developed as a modification intended to reduce valve slippage and improve long-term continence [4,5]. Following the introduction of restorative proctocolectomy with ileal pouch-anal anastomosis (IPAA), the use of continent ileostomies declined substantially [1]. These procedures are now performed only at highly specialized centers and are therefore relatively uncommonly encountered [1]. As such, many contemporary providers may have limited familiarity with their anatomy and management.

We present the case of a patient with a BCIR who accidentally retained a segment of irrigation tubing within the pouch during routine catheterization. Initial endoscopic evaluation was deferred because of concern regarding the small caliber and atypical external appearance of the continent stoma. The patient ultimately underwent endoscopic retrieval of the foreign body through the stoma without difficulty or complication in the operating room.

This case highlights the importance of familiarity with continent ileostomy anatomy and demonstrates that endoscopic access through a BCIR stoma can be successfully achieved and may represent a feasible management option in appropriately selected patients. As these reservoirs are performed very selectively, limited provider exposure may contribute to uncertainty in management and delays in care. This report is not intended to suggest that providers should perform procedures outside their comfort zone or expertise, but rather to emphasize the importance of recognizing altered surgical anatomy and understanding available management approaches.

## Case presentation

A 61-year-old man with a history of ulcerative colitis who had undergone a total abdominal colectomy with creation of a BCIR 13 years ago presented to the emergency department, reporting he had lost a portion of irrigation tubing within his pouch after it accidentally detached while performing his routine irrigation several hours ago. Before his presentation, he had no significant issues with his continent ileostomy. He was otherwise feeling well with no other complaints. His vital signs were within normal limits. Physical exam noted some mild abdominal distension with what appeared to be a "retracted stoma" present in the right lower quadrant that appeared patent and healthy. His labs, including a complete blood count (CBC) and basic metabolic panel (BMP), were within normal limits. Non-contrasted CT imaging demonstrated the known tubing within the pouch approximately 4 cm deep to the stoma. Gastroenterology was consulted for consideration of endoscopic removal of the foreign body.

**Figure 1:**
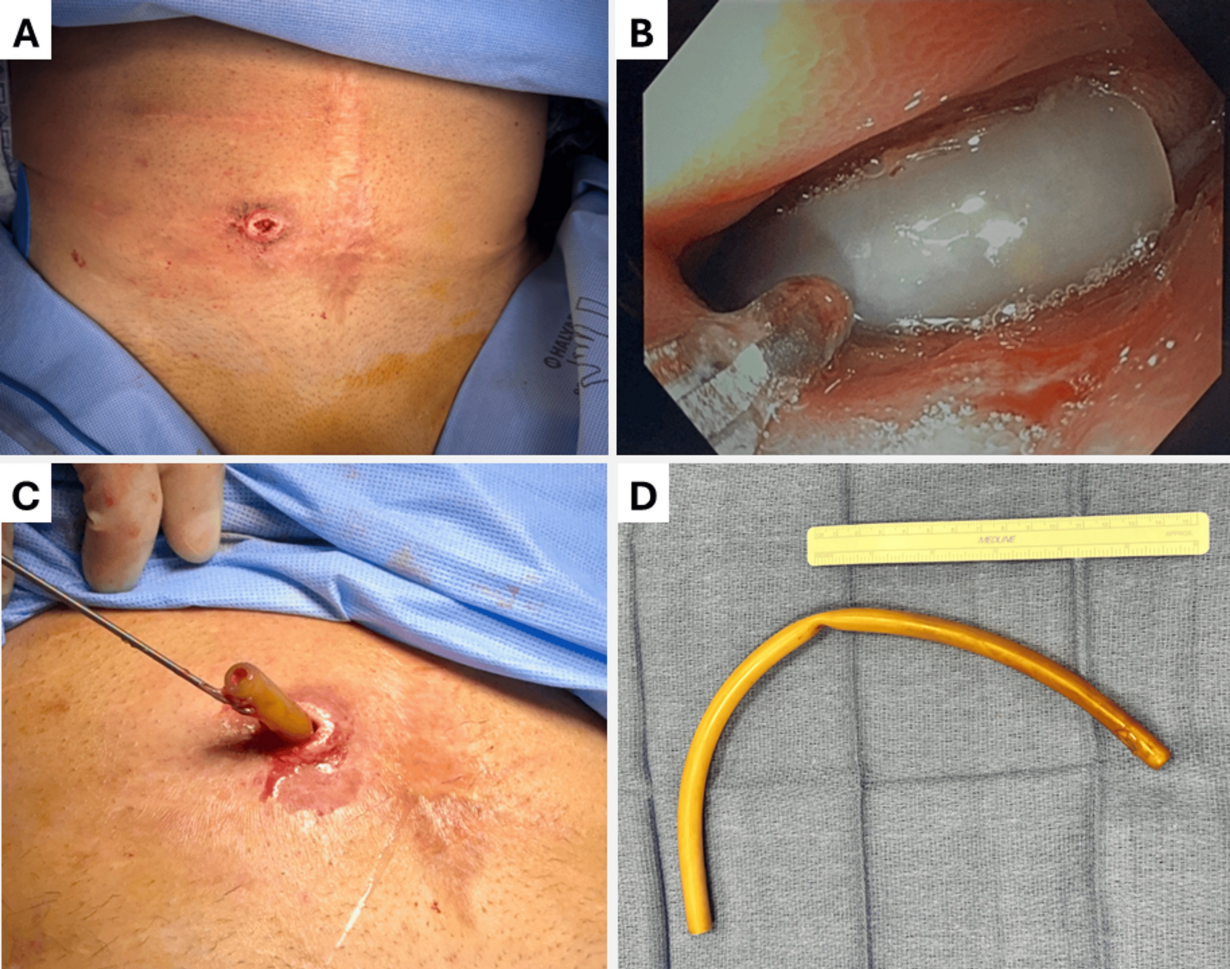
Endoscopic retrieval of retained irrigation tubing within the Barnett continent ileostomy reservoir (A) Continent reservoir stoma appreciated in the RLQ. (B) Endoscopic visualization of the retained irrigation tubing within the BCIR. (C-D) Successful retrieval and removal of the retained, intact irrigation tubing, approximately 30 cm in length, through the stoma. RLQ: right lower quadrant; BCIR: Barnett continent ileostomy reservoir

He was taken to the endoscopy suite for ileoscopy; however, upon examination of the abdomen, the endoscopist was concerned that the stoma was too small to access and the scope was not inserted (Figure 1A). Surgery was then consulted for further evaluation. Informed consent was obtained for endoscopic foreign body removal, and the patient was taken to the operating room and underwent laryngeal mask intubation. The surgeon first attempted digital examination of the stoma, which was unremarkable without appreciable stricture or stenosis. The endoscope was then introduced through the stoma and advanced to the pouch, where the irrigation tubing was clearly visualized (Figure 1B). An endoscopic grasper was used to remove approximately 30 cm of freely mobile irrigation tubing (Figure 1C-1D). No significant mucosal trauma was noted during the procedure. The patient tolerated the procedure well with no complications, and he was discharged home from the recovery unit.

## Discussion

Patients undergoing total proctocolectomy, most frequently for ulcerative colitis or familial adenomatous polyposis, generally have three reconstructive options: conventional (Brooke) ileostomy, ileal pouch-anal anastomosis, and continent ileostomy. Each option carries distinct advantages and limitations with respect to quality of life, continence, and long-term complications [1,2]. Among these, continent ileostomies are encountered least frequently in contemporary surgical practice; the Kock pouch remains the most widely performed continent ileostomy [6].

Briefly, the Kock pouch involves the creation of a nipple valve via retrograde intussusception of the efferent limb into the pouch body, kept in place via several rows of staples [7]. The fundus of the pouch can be anchored to the base of the exit conduit, which is brought through the abdominal wall and anchored to the skin [7]. The pouch is continuously catheterized for several weeks for healing, after which, the patient will then insert an irrigation catheter through the stoma several times a day to empty the pouch at a time and setting of their choosing. Most complications related to Kock pouches are related to the valve, including slippage, prolapse, stricture, and fistula formation [2,8,3]. The BCIR was created as a variant of the Kock pouch, in which a loop of the ileum is wrapped around the pouch outlet to augment the continence of the ileostomy and prevent some of these valve-related complications [4,5,9]. As these procedures are performed only at highly specialized centers, many current providers may have limited familiarity with their anatomy and management [1].

This case highlights an unusual complication of a BCIR involving retention of irrigation tubing during routine pouch catheterization. Although the patient was clinically stable, the altered anatomy and atypical appearance of the continent stoma initially created uncertainty regarding safe endoscopic access. During the initial evaluation, ileoscopy was deferred because of concern that the stoma caliber was too small to safely accommodate the endoscope. However, subsequent surgical evaluation demonstrated a patent stoma without evidence of stenosis or stricture, allowing uncomplicated endoscopic access to the pouch. The continent ileostomy stoma differs from the conventional ileostomy in several important ways. While the conventional ileostomy is intentionally constructed to protrude from the skin via the Brooke technique, continent ileostomies are created flush with the skin surface, making them generally smaller and flatter [10,11]. Because of the internal nipple valve incorporated into the continent ileostomy, there is no need for the use of an external appliance, and often patients will simply cover their stoma with a gauze pad or bandage [11].

Despite the difference in external appearance of the stoma, ileoscopy performed by experienced providers familiar with the relevant anatomy is safe in patients with continent ileostomies. The most recent consensus guidelines from the Global Interventional Inflammatory Bowel Disease Group do not recommend routine surveillance of the pouch for these patients; however, endoscopy can be both diagnostic and therapeutic in the management of pouch-related complications [12-14]. In this patient, CT imaging successfully localized the retained tubing within the pouch to the stoma, helping guide intervention. Careful digital examination before instrumentation confirmed the absence of obstruction or narrowing, after which the endoscope was carefully advanced through the stoma, and the retained tubing was visualized and removed using an endoscopic grasper. Successful retrieval avoided the need for operative exploration.

Management of foreign bodies within continent ileostomy reservoirs is rarely discussed in the literature. The majority of instances described are related to bezoar formation secondary to structural or functional obstruction of the pouch outlet [15,16]. Guidelines recommend endoscopic removal of bezoars when feasible, as well as consideration of the use of a gastroscope as opposed to a colonoscope due to its small caliber and flexibility [15,14]. Though routine surveillance endoscopy is not recommended for BCIR, when endoscopy is performed, evaluation of the entire pouch is recommended, including the afferent limb, inlet, pouch body, nipple valve, and exit channel [14]. To our knowledge, there are no published reports describing the retrieval of other retained foreign bodies from continent ileostomy reservoirs. However, given that the management of continent ileostomies requires repeated catheterization and irrigation of the reservoir, inadvertent retention of irrigation equipment may occur. The frequency of this complication remains unknown, and to our knowledge has not been previously reported.

## Conclusions

This case underscores the importance of recognizing altered surgical anatomy and maintaining familiarity with less common reconstructive procedures. As continent ileostomies are now performed infrequently, many surgeons, gastroenterologists, and emergency providers may have limited exposure to these reservoirs, potentially contributing to delays in diagnosis or management. Multidisciplinary collaboration may therefore be particularly valuable when evaluating patients with uncommon surgical reconstructions. Importantly, this report is not intended to encourage providers to perform procedures outside their expertise, but rather to emphasize that endoscopic access through a BCIR stoma may be safely achievable in selected patients by experienced providers. Increased awareness of continent ileostomy anatomy and available management strategies may help facilitate timely diagnosis and effective treatment in similar presentations.
